# The Nature of the Passive Layer (and Spalled Corrosion Products) on Nonferrous Alloys in Aqueous Corrosive Media—Editorial

**DOI:** 10.3390/ma15062178

**Published:** 2022-03-16

**Authors:** Herman Potgieter

**Affiliations:** Department of Natural Science, Manchester Metropolitan University, Manchester M15 6BH, UK; h.potgeiter@mmu.ac.uk

Mild steel and various stainless steels are the workhorse alloys in construction and process industries, used in a myriad of applications. However, equally important are alloys and materials such as those of aluminium, magnesium, titanium, tantalum, zirconium and various combinations thereof with one another and other alloying additions. Equally varied are the corrosive environments in which these alloys and combinations are applied to limit degradation and supply solutions to their applications and maintenance. Despite this, corrosion remains a multibillion-dollar phenomenon worldwide.

Corrosion is a surface phenomenon and process which occurs between a material/alloy and its corrosive environment. As such, any structure, formed film or corrosion product on the surface of a material in contact with its corrosive environment is important, and plays a role in the subsequent degradation or reduced degradation of the material in question. It is well known and widely accepted that the corrosion resistance of stainless steels, titanium alloys and aluminium alloys depends greatly on the spontaneous formation of passive oxide films on their surfaces.

Equally as important, but much less investigated than ferrous-based alloys, are the surface films of other alloys and materials not comprising ferrous-alloy groups, e.g., magnesium, titanium, aluminium and several other types of alloys. These films can be the result of exposure to the atmosphere and the subsequent oxide film formation, or can occur as the result of an inhibited or noninhibited corrosion process. Whether continuous or not, all such formed surface films obviously affect the material’s degradation and corrosion behaviour and degree of destruction. This Special Issue, which I am honoured to edit as a Guest Editor, aims to highlight and describe the nature of surface films and corrosion products occurring/forming as a result of the exposure of nonferrous metals to all sorts of corrosive environments and their consequent influence on the corrosion behaviour of such alloys in the corrosive environments where they are applied, be it inside the human body or under process conditions.

If you work with any such alloys or materials, it is my great pleasure to invite you to submit your work for publication to this Special Issue of *Materials*. With its high impact factor of 3.623, your work in this open access publication is sure to receive wide coverage and attraction. As you can see from the papers already published, they are from highly reputable research groups and laboratories.

## Data Availability

Not applicable.

